# Timing of vasoactive agents and corticosteroid initiation in septic shock

**DOI:** 10.1186/s13613-022-01021-9

**Published:** 2022-05-30

**Authors:** Mahmoud A. Ammar, Abdalla A. Ammar, Patrick M. Wieruszewski, Brittany D. Bissell, Micah T. Long, Lauren Albert, Ashish K. Khanna, Gretchen L. Sacha

**Affiliations:** 1 Department of Pharmacy, Yale New Haven Health System, 20 York Street, New Haven, CT 06510 USA; 2 Departments of Anesthesiology and Pharmacy, Mayo Clinic, 200 First Street SW, Rochester, MN USA; 3 Department of Pulmonary, Critical Care, and Sleep Medicine, College of Medicine, University of Kentucky, Lexington, KY USA; 4 Department of Pharmacy Practice and Science, College of Pharmacy, University of Kentucky, Lexington, KY USA; 5 Department of Anesthesiology, University of Wisconsin Hospitals and Clinics, 600 Highland Ave, Madison, WI USA; 6 Department of Pharmacy, Hospital of the University of Pennsylvania, 3400 Spruce Street, Philadelphia, PA USA; 7 Department of Anesthesiology, Section on Critical Care Medicine, Wake Forest School of Medicine, Wake Forest Center for Biomedical Informatics, Perioperative Outcomes and Informatics Collaborative, Medical Center Boulevard, Winston-Salem, NC USA; 8 Outcomes Research Consortium, Cleveland, OH USA; 9 Department of Pharmacy, Cleveland Clinic, 9500 Euclid Avenue, Hb-105, Cleveland, OH USA

**Keywords:** Sepsis, Septic shock, Resuscitation fluids, Vasoactive agents, Catecholamines, Vasopressin, Angiotensin II, Corticosteroids

## Abstract

Septic shock remains a health care concern associated with significant morbidity and mortality. The Surviving Sepsis Campaign Guidelines for Management of Sepsis and Septic Shock recommend early fluid resuscitation and antimicrobials. Beyond initial management, the guidelines do not provide clear recommendations on appropriate time to initiate vasoactive therapies and corticosteroids in patients who develop shock. This review summarizes the literature regarding time of initiation of these interventions. Clinical data regarding time of initiation of these therapies in relation to shock onset, sequence of treatments with regard to each other, and clinical markers evaluated to guide initiation are summarized. Early-high vasopressor initiation within first 6 h of shock onset is associated with lower mortality. Following norepinephrine initiation, the exact dose and timing of escalation to adjunctive vasopressor agents are not well elucidated in the literature. However, recent data indicate that timing may be an important factor in initiating vasopressors and adjunctive therapies, such as corticosteroids. Norepinephrine-equivalent dose and lactate concentration can aid in determining when to initiate vasopressin and angiotensin II in patients with septic shock. Future guidelines with clear recommendations on the time of initiation of septic shock therapies are warranted.

## Introduction

Sepsis and septic shock remain major health care problems associated with significant morbidity and mortality [1, 2]. Surviving Sepsis Campaign (SSC) Guidelines for Management of Sepsis and Septic Shock recommend early initiation of fluids, broad-spectrum antimicrobials, and in patients with septic shock, vasopressors with norepinephrine as the recommended first-line agent [3]. The inflammatory status of septic shock patients results in vascular endothelial damage and shedding of the endothelial glycocalyx, leading to increased permeability, microcirculatory dysfunction, and vasodilation [4]. Resuscitation fluids remain the first-line therapy for mitigating hemodynamic compromise in septic shock by restoring intravascular volume, cardiac output, and oxygen delivery. However, the effect of resuscitation fluids dissipates within 30–60 min, limiting repeated benefit when more disease-directed therapies, such as vasopressors and antimicrobials, are available [5–8]. The importance of prompt initiation of antimicrobial therapy in sepsis and septic shock patients has been well established, and a strong association with worse outcomes with each hour of delay in antibiotic initiation for septic shock patients exists [9, 10]. As such, initiation of antibiotics within the first hour of definite or probable sepsis identification is imperative [3, 11, 12].

Beyond the initial management of septic shock with fluids and antimicrobial therapy, the SSC guidelines do not provide clear recommendations on the appropriate time to initiate vasoactive agents and corticosteroids, resulting in practice variations [13–15]. Clinicians treating patients recognize inter-patient variability in response to septic shock therapies, making it challenging to have generalized recommendations. Despite this variability, clinicians tend to be interested in a protocolized approach in managing septic shock patients [11, 16].

**Fig. 1:**
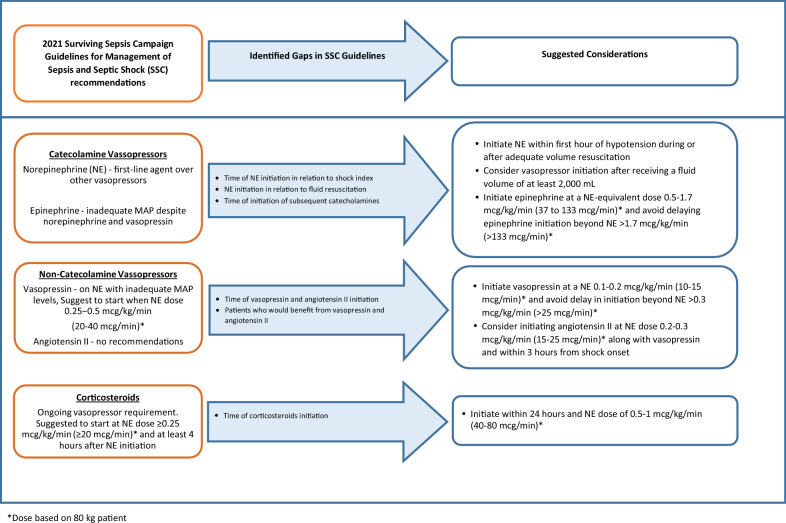
Identified gaps in the Surviving Sepsis Campaign Guidelines for Management of Sepsis and Septic Shock (SSC) regarding initiation of vasoactive therapies and corticosteroids and suggested considerations for appropriate initiation of therapies

Current literature describes the timing of initiation of interventions temporally from shock onset and the sequence of therapies in relation to each other. Clinical markers, including norepinephrine dose requirements or lactate concentrations, have also been suggested to guide time of initiation. This narrative review summarizes literature surrounding the time of initiation of vasopressor therapy and corticosteroids, describes current gaps in the literature, and considerations for initiation of these therapies (Fig. 1). We aim to provide guidance to bedside clinicians to help appropriately timed interventions to decrease time spent in hemodynamic compromise.

For this review, a PubMed English-language literature from January 2000 to January 2022, including the following terms: norepinephrine, epinephrine, arginine vasopressin, angiotensin II, corticosteroids, fluid resuscitation, septic shock, and sepsis, were taken into consideration. Relevant clinical data, including controlled trials, observational studies, review articles, guidelines, and consensus statements, were narratively summarized, focusing on specific controversial questions regarding the initiation of these therapies in patients with septic shock. Even though the level of evidence of identified citations was low, there was a trend demonstrating a consistent interest in reporting the appropriate time of initiation of septic shock-related therapies.

### Time of vasopressor therapy initiation

Current SSC guidelines recommend norepinephrine as first-line vasopressor agent in septic shock [3]. When goal mean arterial pressure (MAP) cannot be achieved, adding vasopressin is suggested rather than increasing norepinephrine dose. Epinephrine is a recommended alternative for patients with inadequate MAP levels despite norepinephrine and vasopressin. While dobutamine can be added to norepinephrine in septic shock patients with cardiac dysfunction and persistent hypoperfusion despite adequate resuscitation. Although dopamine has previously served a primary role in correcting hypotension, the evidence has been contrary, with signals of increased mortality in a meta-analysis compared to norepinephrine [17]. This, coupled with the excessive beta stimulation in the myocardium with high dosages of dopamine leading to undesirable arrhythmias, has limited the application of dopamine in septic shock to settings where other catecholamines (norepinephrine or epinephrine) are unavailable [18] 19. While guidelines strongly recommend using norepinephrine as the first-line vasoactive agent in septic shock, there is limited data comparing available second-line agents. Therefore, an individualized approach based on vasoactive therapy pharmacology, desired pharmacodynamic actions, and patient characteristics should be used to decide on a vasoactive therapy regimen [3, 14].

While the true definition of what constitutes ‘high dose’ catecholamines remains a matter of debate, there is evidence of harm associated with norepinephrine monotherapy over a range of supra-therapeutic doses [20]. The risks of vasopressor therapy must be considered and the lowest effective dose should be utilized as several evaluations have shown that higher catecholamine doses, especially those exceeding 0.5–1 mcg/kg/min, are independently associated with adverse effects and mortality [21–23]. A retrospective study assessing survival in septic shock patients requiring high-dose vasopressors reported that the weight-based mean dose of vasopressor that was associated with increased mortality (with a 73% sensitivity and 74% specificity) was > 0.75 mcg/kg/min [24]. Therefore, a basic tenet of early multi-modal vasopressor therapy emphasizes using multiple vasopressors early on in the timeline of shock and not necessarily after declaration of failure of a certain vasopressor. Here, less catecholamines and a more adjunctive therapy environment would be deemed beneficial [25]. It is important to note that even for specific vasopressors such as norepinephrine, there is inconsistency in the literature on how doses are reported. Collectively, studies have reported doses utilizing norepinephrine tartrate, norepinephrine base, and norepinephrine hydrochloride interchangeably. These products carry different norepinephrine base doses per unit, which adds to the complexity of reporting optimal doses to initiate vasoactive agents [26].

A critical aspect of early vasopressor initiation in septic shock is the interplay with adequate fluid resuscitation. Vasopressor use and fluid administration were evaluated in the Characterization of Vasopressor Requirements in Shock (CHASERS) study [27]. In this multicenter, prospective, observational cohort, results showed an increased odds of 30-day in-hospital mortality within the first 6 h of shock onset with increasing vasopressor dosing intensity (VDI), defined as the total vasopressor dose infused across all vasopressors in norepinephrine equivalents. With increasing VDI, higher mortality rates remained through the first 24 h, where the median vasopressor dose was 8.5 mcg/min norepinephrine equivalents (equating to 0.1 mcg/kg/min in an 80 kg patient). Interestingly, receiving a fluid volume of at least 2000 mL attenuated the association between VDI and increased mortality. Additionally, early-high VDI, defined as vasopressor initiation within the first 6 h and VDI ≥ 15 mcg/min norepinephrine equivalents, was associated with lower mortality than early-low VDI (VDI < 15 mcg/min norepinephrine equivalents within the first 6 h), late-high VDI (VDI ≥ 15 mcg/min norepinephrine equivalents after 6 h and through hour 24), and sustained VDI exposure (VDI ≥ 15 mcg/min norepinephrine equivalents during the first 6 h and remained elevated for the entire 24 h period), further supporting early vasopressor initiation [27]. Although this study did not evaluate weight-based dosing of vasopressors, 15 mcg/min of norepinephrine equivalents can be equated to roughly 0.2 mcg/kg/min in an 80 kg patient. Importantly, all these analyses in the CHASERS study were adjusted for severity of illness factors such as APACHE III score, comorbidities, and other resuscitative interventions. In contrast, a recently published prospective, multicenter, observational study classified patients into early and late vasopressor groups based on vasopressor timing in relation to fluid resuscitation [28]. In this propensity-matched analysis, there was a greater risk of 28-day mortality when vasopressor initiation occurred within 1 h after fluid bolus administration (47.7% vs. 33.6%; *p* = 0.013). While both groups received more than 30 mL/kg of fluid, a greater volume was administered to the late vasopressor group within 6 h of shock identification (33.4 ± 21.0 vs. 38.0 ± 15.7 mL/kg; *p* = 0.046). Vasopressor dosing is not reported in this study, limiting the comparison to previously mentioned publication [28]. Future studies are needed to assess the relationship between fluid and vasopressor initiation and restrictive fluid strategies in sepsis. While the threshold for ideal fluid resuscitation cannot be concluded at this time, appropriate intravascular resuscitation appears to diminish the relationship between vasopressor dose and mortality.

Balancing the need between vasopressors and limiting catecholamine dosages, draws to question the timing of vasopressor initiation in patients with septic shock and persistent hypotension. Hypotension in critically ill patients with septic shock strongly correlates with mortality and organ system injury [29, 30]. Regardless of the concerns with excessive catecholamine dosages, norepinephrine initiation is fundamental in septic shock to achieve MAP goals and restore perfusion to vital organs. When delayed, especially beyond 6 h, patients appear to require vasopressors for longer durations, resulting in an unresolved shock [27]. Identifying the exact time to initiate vasopressors is challenging. It has been suggested that low diastolic arterial pressure (DAP) should reflect the loss of vascular tone and systemic vasodilation. However, DAP is not traditionally incorporated into the definition of septic shock and its utility warrants further investigation [31]. A study evaluating the ratio between DAP and heart rate (diastolic shock index, DSI) before and at vasopressor initiation reported that the increase of DSI was associated with an increased risk of death and that DSI could be utilized to guide the appropriate time to initiate vasopressor therapy [32].

Early and aggressive vasopressor initiation should be considered and is supported by multiple studies noting an association between delayed therapy and increased mortality [21, 27, 33, 34]. These findings are consistent with the 2018 SSC hour-1 bundle, which recommends vasopressor therapy within the first hour during or after volume resuscitation [35]. One small retrospective study found that appropriate early antibiotics and achieving adequate global perfusion, but not liberal vasopressor therapy, was associated with improved organ function [36]. However, subsequent retrospective analyses found that vasopressor therapy delays worsen clinical outcomes, including mortality [33, 34]. One evaluation found that each hour delay in norepinephrine initiation resulted in a 5.3% increase in mortality [34]. Given the limitations of prior retrospective studies, the CENSER trial, a single-center, prospective, double-blind, placebo-controlled trial, was published in 2019 and evaluated shock control rate in patients randomized to early low-dose norepinephrine administration or placebo [37]. Shock control rate was defined as a sustained mean arterial pressure of at least 65 mmHg with evidence of adequate perfusion. Early vasopressor group received norepinephrine at 1.5 h compared to 3 h in the standard treatment group. Shock control at 6 h was met in 76.1% of patients in the early vasopressor group compared to 48.4% in the standard group (*p* < 0.001). While there was no difference in 28-day mortality, early norepinephrine group had a lower rate of cardiogenic pulmonary edema and new-onset arrhythmias [37]. Additionally, early vasopressors could limit the harmful effect of positive fluid balance in septic shock by potentiating the effect of fluids and avoiding fluid overload. In a retrospective study in septic shock patients, patients who received norepinephrine within the first 2 h of resuscitation received less fluids than those who received delayed vasopressors [34]. Norepinephrine should be initiated early, ideally within 1 h of shock onset, and post adequate fluid resuscitation.

### Time of adjunctive vasopressor therapy initiation

#### Vasopressin

Vasopressin is a non-catecholamine vasoactive agent with pharmacologic activity at vasopressin-1 (V1) receptor, causing vasoconstriction, and V2-mediated antidiuretic activity. The landmark Vasopressin in Septic Shock Trial (VASST) compared the utilization of vasopressin and norepinephrine to norepinephrine alone [38]. Overall, no difference in 28-day mortality was detected between groups (35.4% vasopressin vs. 39.3% norepinephrine alone). However, subgroup analyses identified a mortality benefit with the use of vasopressin in less severe septic shock patients, who were those with a norepinephrine dose at randomization ≤ 15 mcg/min and those with a lactate concentration at randomization of  ≤ 1.4 mmol/L. Subsequently, a single-center, prospective, open-label trial of early vasopressin initiated within the first four hours of norepinephrine showed faster achievement and maintenance of goal MAP compared to norepinephrine monotherapy [39]. Achievement of goal MAP and reduction in catecholamine dose requirements has been a consistent finding with the use of vasopressin and has also been associated with improved outcomes [38, 40]. One recent retrospective observational evaluation found that after adjustment for Sequential Organ Failure Assessment (SOFA) score, Acute Physiology and Chronic Health Evaluation.

(APACHE) III score, and catecholamine dosage, achieving a positive hemodynamic response (occurring in 45.4% of vasopressin recipients) 6 h after vasopressin initiation was independently associated with reduced odds of intensive care unit (ICU) mortality (odds ratio (OR), 0.51; 95% CI 0.35–0.76) [41]. These findings suggest that early vasopressin initiation may be beneficial and target hemodynamic response for its continued utilization.

VASST showed reduced mortality in the vasopressin group when the norepinephrine dose at randomization was ≤ 15 mcg/min compared to receipt of norepinephrine alone. Observational analyses evaluating only vasopressin recipients have shown similar trends. One evaluation found that norepinephrine-equivalent dose at vasopressin initiation was independently associated with increased odds of ICU mortality (OR, 3.14; 95% CI 1.36–7.28) [41]. Additionally, a recent evaluation found that after adjustment for severity of illness covariates, including SOFA score and APACHE III score, odds of in-hospital mortality increased 20.7% for every 10 mcg/min increase in norepinephrine-equivalent dose under 60 mcg/min at the time vasopressin initiation (OR, 1.21; 95% CI 1.09–1.34) [42]. No association was detected when norepinephrine-equivalent dose exceeded 60 mcg/min at vasopressin initiation. Regarding the clinical marker lactate, data reveal an association with increased odds of ICU mortality depending on timing of vasopressin initiation (OR, 1.10; 95% CI 1.04–1.18) [41]. One evaluation found an 18% increase in the odds of in-hospital mortality for each mmol/L increase in lactate concentration when vasopressin was initiated at 12 h from shock onset [42]. Contrastingly, the VASST study and aforementioned retrospective evaluations did not detect associations between the time, in hours, from shock onset to vasopressin initiation and clinical outcomes [38, 41, 42]. These data indicate that when utilized, vasopressin should be initiated when patients are on low norepinephrine-equivalent doses or have low lactate concentrations rather than delaying therapy until significant elevations in clinical markers indicating more severe illness. The benefit of early vasopressin may be due to its mechanism involving endocrine replacement, as vasopressin levels are known to decrease after hypotension onset rapidly [36–38]. A second rationale may be its norepinephrine-sparing ability, limiting the immunomodulatory effects and exposure to norepinephrine [43]. Regardless, efforts should be made to initiate vasopressin early rather than delaying therapy.

Norepinephrine-equivalent dose and lactate concentration are important markers that can be utilized to aid in determining when to initiate vasopressin in septic shock patients. However, at this time, it is unknown if one is more predictive than the other or how to utilize both markers together. The 2021 iteration of the SSC guidelines support these themes and suggest vasopressin as an option to be added to norepinephrine in patients not at goal MAP rather than escalating the dose of norepinephrine [3]. Clinically, at the bedside, particularly if lactate concentration is not readily available or has not recently been drawn, it seems norepinephrine-equivalent dose may be the more accessible marker to utilize and vasopressin initiation should be considered before doses exceed 10–15 mcg/min (0.1–0.2 mcg/kg/min in an 80 kg patient).

#### Angiotensin II

Angiotensin II is an octapeptide produced by cleavage of angiotensin I by the angiotensin-converting enzyme (ACE) and has high affinity for angiotensin II type 1 receptor [44]. Stimulation of this G-protein coupled receptor on peripheral vascular smooth muscle results in aldosterone secretion, endogenous vasopressin release, and direct arterial and venous vasoconstriction [44]. A synthetic analogue of this human peptide has been evaluated in the Angiotensin II for Treatment of High-Output Shock (ATHOS-3) study. In this phase 3 study, patients had refractory shock requiring a minimum of 0.2 mcg/kg/min norepinephrine-equivalents with a median of 0.34 mcg/kg/min at time of enrollment [45, 46]. Subsequently, early post-marketing evaluations have demonstrated clinical adoption of angiotensin II to be incongruent with ATHOS-3. These studies included all-comers receiving angiotensin II for vasodilatory shock, with most patients having septic shock. The background vasopressor requirement at the time of angiotensin initiation was 0.58 mcg/kg/min and 0.55 mcg/kg/min, far greater than the phase 3 trial [47, 48].

In ATHOS-3, 69.9% of patients who received angiotensin II achieved the primary hemodynamic endpoint compared to 23.4% of recipients receiving standard of care (OR, 7.95; 95% CI 4.76–13.3, *p* < 0.001) [46]. Similar hemodynamic response rates were found in post-marketing evaluations by Wieruszewski et al. (67%) and Smith et al. (80.1%), although definitions for response varied [47, 48]. However, in a responder analysis, those that had a positive hemodynamic response to angiotensin II were less likely to die at 30 days as compared to a non-response (hazard ratio (HR), 0.50; 95% CI 0.35–0.71, *p* < 0.001), despite baseline severity of illness [47]. Additionally, patients with lower serum lactate concentrations were more likely to have a positive hemodynamic response to angiotensin II (OR, 1.11 per mmol/L; 95% CI 1.05–1.17, *p* < 0.001) and survive at 30 days (mortality HR, 0.94 per mmol/L; 95% CI 0.91–0.96, *p* < 0.001) [47]. When dichotomized by baseline vasopressor requirement, angiotensin II recipients in ATHOS-3 were more likely to achieve blood pressure targets if their baseline vasopressor requirement was lower (< 0.5 mcg/kg/min) [46]. Considering baseline vasopressor requirements in the post-marketing environment, an even greater vasopressor sparing effect is noted. This was more pronounced in those requiring < 0.2 mcg/kg/min (mean difference at 3 h, − 97.7%; 95% CI − 171.7 to −23.8%, *p* = 0.01), however findings still held true at < 0.3 mcg/kg/min, albeit to a lesser extent (mean difference at 3 h, − 68.3%; 95% CI − 133.5 to − 3.0%, *p* = 0.04) [48]. These data suggest the application of angiotensin II has the greatest chance of success early and positively affects outcomes when shock is less severe.

In the ATHOS-3 study, angiotensin II recipients experienced a more remarkable change in background vasopressor dosage at 3 h compared to placebo (− 0.03 vs. + 0.03 mcg/kg/min, *p* < 0.001) [46]. Wieruszewski et al. found a more significant vasopressor sparing effect with angiotensin II responders experiencing a change of − 0.20 mcg/kg/min in background vasopressor dose at 3 h [47]. Despite a majority receiving vasopressin (*n* = 248, 92%), those already receiving vasopressin were more likely to have a favorable hemodynamic response to angiotensin II in multivariable analysis (OR, 6.05; 95% CI 1.98–18.6, *p* = 0.002). Similarly, Smith et al. found a mean difference of − 0.16 mcg/kg/min vasopressor dose at 3 h [48]. Whether these differences and greater vasopressor sparing effect are due to a higher baseline vasopressor requirement or differences in titration schema and target blood pressure goals remains unclear.

There appears to be a subset of patients with derangement in endogenous renin–angiotensin system function that experience a marked response to angiotensin II and may benefit from earlier administration. When there is a defect or insufficiency in ACE, endogenous angiotensin II is not produced, leading to angiotensin I and renin accumulation [49]. This increases the amount of substrate available for degradation by neprilysin and ACE-2, leading to accumulation of vasodilatory angiotensin byproducts, namely angiotensin 1–9 and angiotensin 1–7 [49]. These substances provide feedback on the juxtaglomerular cells to produce additional renin, further potentiating vasodilatory pathways. Accordingly, patients from the ATHOS-3 study with high renin (i.e., excess angiotensin I compared to angiotensin II) have a profound death-sparing effect when administered angiotensin II compared to placebo (28-day survival 70% vs. 51%, HR, 0.56; 95% CI 0.35–0.88, *p* = 0.01) [50]. Therefore, suppressing this catastrophic negative feedback loop and preventing massive buildup of vasodilatory mediators by exogenous angiotensin II administration may serve as a mechanism to improve outcomes in septic shock.

#### Epinephrine

The exact dose and timing of escalation to adjunctive catecholamine agents are not well elucidated, which includes epinephrine. As previously stated, epinephrine is often considered in tissue hypoperfusion and reduced cardiac output and may be preferred over vasopressin in patients with mixed cardiogenic shock due to its beta-receptor action and lack of inotropic support with vasopressin [51]. A novel dose-finding study examined the optimal norepinephrine-equivalent dose at which epinephrine was initiated in patients with septic shock [52]. This study identified the optimal norepinephrine-equivalent dose range between 37 and 133 mcg/min to initiate epinephrine, 0.5–1.7 mcg/kg/min in an 80 kg patient. In this dose range, 29% of patients achieved hemodynamic stability with the initiation of epinephrine compared to 15% of patients who had epinephrine initiated outside of this dose range (*p* = 0.03). Based on these data, a norepinephrine-equivalent dose of 37 to 133 mcg/min was proposed as an ideal breakpoint for starting epinephrine, and delaying epinephrine administration to norepinephrine doses exceeding 133 mcg/min should be avoided. Delaying therapy may impact achievement of hemodynamic stability and can be futile [52]. Following initiation of norepinephrine, escalation to epinephrine at previously described norepinephrine-equivalent doses can be considered concurrently with catecholamine-sparing strategies. As data regarding the optimal timing of additional adjunctive catecholamine agents are not available, thoughtful escalation is imperative to avoid excessive catecholamine exposure. When utilizing epinephrine, adverse events should be monitored, including tachyarrhythmias, hyperglycemia, hypokalemia, and hyperlactatemia.

### Time of non-catecholamine vasopressors

Following first-line norepinephrine application, choice of and timing of secondary, non-catecholamine vasopressors remain unclear due to restraints of clinical trial methodology [38, 46] and extrapolation of retrospective cohort studies to clinical practice [41, 47]. Regardless, excessive catecholamine exposure increases risk of arrhythmias, critical organ damage, and tissue ischemia [53]. High vasopressor doses and significant cumulative exposures are associated with worse outcomes in septic shock [54]. Even among angiotensin II recipients, those with greater norepinephrine requirements are more likely to die (HR, 1.61 per 1 mcg/kg/min; 95% CI 1.03–2.51 *p* = 0.037) [47]. Therefore, minimizing exposure to catecholamine vasopressors with vasopressin and angiotensin II may serve as an additional mechanism to improve outcomes in septic shock and reduce time under inadequate perfusion pressure [43].

Norepinephrine-equivalent dose can be utilized to determine when to initiate vasopressin and angiotensin II in patients with septic shock [46]. As described previously, vasopressin initiation should be considered before doses exceed 10–15 mcg/min (0.1–0.2 mcg/kg/min in an 80 kg patient) [42]. Similarly, angiotensin II should follow rapidly when hemodynamic stability is not achieved after vasopressin, given data suggesting a synergistic effect. This addition might be made before norepinephrine doses exceed 15–25 mcg/min (0.2–0.3 mcg/kg/min in an 80 kg patient), again sparing catecholamine toxicity [47].

As the molecular complexity of various shock endotypes continues to grow, there is a need to establish a role for biomarker-guided non-catecholamine vasopressor initiation to truly individualize timely resuscitation [55]. Contenders that have emerged as potential targets include plasma vasopressin concentrations and direct renin concentrations. Although in retrospective settings, a strong relationship between plasma vasopressin concentrations in shock and favorable hemodynamic response to vasopressin has not been demonstrated, there is still an opportunity to identify profoundly vasopressin-deficient endotypes due to rapid vasopressin plasma clearance mechanisms [56, 57]. On the other hand, renin has persistently outperformed lactate in predicting hospital and ICU mortality in patients with hypotension [58, 59]. In addition to the strong relationship between hyper-reninemia and favorable response to angiotensin II, renin is quickly evolving as a promising prognosticator in shock. Coupling these sensitive and specific biomarkers, ideally through the development of point-of-care assays, along with clinical characteristics of the presenting shock case, will be crucial for the future of rapid sepsis care.

### Time of corticosteroid therapy initiation

The hyper-inflammatory state of septic shock leads to vasodilation and hypotension. Corticosteroids are used in the management of septic shock for their anti-inflammatory properties through inhibition of nuclear factor-KB, thus reducing interleukin (IL)-1, IL-6, IL-8, tumor necrosis factor (TNF)-α, and TNF receptors 1 and 2 [60]. Additionally, corticosteroids inhibit nitric oxide (NO) synthase, inhibiting sepsis-induced NO-mediated vasodilation [61]. Lastly, exogenous corticosteroids address insufficient cortisol levels, mitigating a relative adrenal insufficiency responsible for further hemodynamic instability in septic shock [62].

The role of corticosteroid therapy in septic shock remains debatable. Controversy exists surrounding benefits such as duration of shock, vasopressor requirements, and mortality, which must be weighed against adverse events including infection, hyperglycemia, and hypernatremia [63–67]. Four large, randomized control trials evaluated the use of corticosteroids in septic shock patients and reported inconsistent findings [64–67]. The Annane et al. study and the Activated Protein C and Corticosteroids for Human Septic Shock (APROCCHSS) trial reported a significant benefit in all-cause mortality in septic shock patients who received a low-dose corticosteroid regimen compared to placebo [64, 67]. In contrast, the Corticosteroid Therapy of Septic Shock (CORTICUS) trial and the Adjunctive Corticosteroid Treatment in Critically Ill Patients with Septic Shock (ADRENAL) trial failed to demonstrate survival benefit [65, 66]. Nevertheless, several trials found that hydrocortisone was associated with a shorter time to shock reversal, supporting its use in septic shock patients with ongoing vasopressor requirements [64–66]. The variation in the inclusion criteria of these landmark trials might have contributed to different outcomes. The Annane et al. study and APROCCHSS trial that demonstrated mortality benefit included more severely ill patients than the CORTICUS and ADRENAL trials [64–67]. In contrast, the CORTICUS and ADRENAL trial did not specify fluid resuscitation and vasopressors for inclusion [64, 67]. As such, it can be argued that the initiation of corticosteroid therapy in septic shock patients should be reserved for patients who are hemodynamically unstable despite adequate fluid resuscitation and vasopressors administration. There is uncertainty around the optimal dose and time course of hydrocortisone therapy mainly due to disparities in the study designs and heterogeneity of results. Historically (pre-1989), studies investigating high-dose, short-duration corticosteroids showed a significant increase in mortality. While more recent studies investigated low doses, prolonged duration demonstrated positive mortality outcomes [68]. The robust studied doses in trials are 200 mg per day of intravenous hydrocortisone in divided doses with therapy durations ranging from 5–7 days [64–67, 69]. The current guidelines recommend intravenous hydrocortisone at a dose of 200 mg per day [18, 70].

The design of these trials differed in time to randomization and vasopressor dose at enrollment. The majority of these trials reported initiation of corticosteroids at norepinephrine mean dose of 0.5–1 mcg/kg/min [64, 65, 67]. The Annane et al. study had the shortest time to enrollment of 8 h [64]. Both ADRENAL and APROCCHSS study designs had a 24-h from shock onset to enrollment, while the CORTICUS trial had the longest time to enrollment of 72 h [65–67]. The Annane et al. and ADRENAL studies were the only trials that reported mean time from shock onset to first steroid dose (4.1 ± 3.4 h and 20.9 ± 91.9 h, respectively) [64, 66]. It has been suggested that the delay in initiation of corticosteroids in the CORTICUS trial contributed to the lack of mortality benefit.

A retrospective cohort study evaluated the appropriate time to initiate corticosteroids after shock onset. Patients (*n* = 1470) were grouped into five different timing cohorts based on time after shock onset: 0–6, 6–12, 12–24, 24–48, or > 48 h [71]. On multivariable linear regression, timing of corticosteroid initiation was independently associated with more days alive and free from vasopressors when comparing initiation within 0–6 h with > 48 h, 6–12 h with > 48 h, and 12–24 h with > 48 h and was associated with reduced ICU mortality when comparing receipt within 0–6 h of shock onset to > 48 h after shock onset [71]. A recent multicenter, propensity score-weighted observational cohort study (*n* = 198) evaluated early (within 12 h of vasopressor initiation) versus late (after 12 h of vasopressor initiation) low-dose corticosteroid initiation in septic shock and identified that early initiation was associated with shorter time to vasopressor discontinuation compared with late (40.7 vs. 60.6 h; *p* = 0.0002) [72].

Although there is no clear recommendation with regard to the time of initiation of corticosteroids in septic shock patients, the SSC guidelines suggest corticosteroids in patients with septic shock and ongoing requirements for vasopressor therapy with initiation suggested as early as 4 h after vasopressor initiation and at norepinephrine dose of at least 0.25 mcg/kg/min [3]. Based on the existing literature and purported mechanisms of benefit, we believe the early initiation of corticosteroid therapy in sepsis, specifically within 24 h of shock, despite adequate fluid resuscitation and vasopressor administration (0.5–1 mcg/kg/min norepinephrine-equivalent dose) is reasonable.

## Conclusion

Septic shock is a complex disorder associated with high mortality. Timely initiation of therapeutic interventions to augment hemodynamics and reverse shock state is imperative. Clinical guidelines lack recommendations on time of initiation of septic shock-related therapies. Norepinephrine initiation after adequate fluid resuscitation may attenuate the association between vasopressor dosing intensity and increased mortality. Early high-dose vasopressor initiation within the first 6 h of shock onset has been associated with lower mortality. Following norepinephrine, the exact dose and timing of escalation to adjunctive vasopressor agents are not well elucidated in the literature. However, recent data indicate that timing may be an important factor in initiating vasopressors and adjunctive therapies. Norepinephrine-equivalent dose and lactate concentration can aid in determining initiation of vasopressin and angiotensin II in septic shock patients. Biomarker-guided angiotensin II use may be facilitated using renin. Vasopressin initiation at a norepinephrine-equivalent dose of 10–15 mcg/min (0.1–0.2 mcg/kg/min in an 80 kg patient) or serum lactate below 2.3 mmol/L has shown to be associated with a mortality benefit. Similarly, observational studies demonstrated that initiating angiotensin II at norepinephrine dose 15–25 mcg/min (0.2–0.3 mcg/kg/min in an 80 kg patient) along with vasopressin and within 3 h from shock onset might be beneficial. Clinicians should consider the addition of epinephrine in patients with a cardiogenic component to their shock state when doses of other vasopressors have been optimized and should not significantly delay initiation of epinephrine. Randomized controlled trials evaluating the use of corticosteroids included septic shock patients who received corticosteroids at a norepinephrine-equivalent dose of 0.5–1 mcg/kg/min within at least 24 h from shock onset. Simplified guidelines with clear recommendations, reflecting real-world applicability, and based on well-designed studies are warranted.

## Data Availability

All data generated or analyzed during this review are included in the published studies and their additional information files.

## Abbreviations

SSC: Surviving sepsis campaign guidelines for management of sepsis and septic shock
MAP: Mean arterial pressure
CHASERS: Characterization of vasopressor requirements in shock study
VDI: Vasopressor dosing intensity
VASST: Vasopressin in Septic Shock Trial
ICU: Intensive care unit
OR: Odds ratio
ACE: Angiotensin-converting enzyme
ATHOS-3: Angiotensin II for treatment of high-output shock
HR: Hazard ratio
IL: Interleukin
TNF-α: Tumor necrosis factor-α
NO: Nitric oxide
APROCCHSS: Activated Protein C and Corticosteroids for Human Septic Shock Trial
CORTICUS: Corticosteroid Therapy of Septic Shock Trial
ADRENAL: Adjunctive Corticosteroid Treatment in Critically Ill Patients with Septic Shock Trial
